# Low dose ultra-slow infusion thrombolytic therapy (LDUSITT) as an alternative option in a COVID-19 patient with free-floating right atrial thrombus: a case report and review of literature

**DOI:** 10.1186/s12959-023-00457-8

**Published:** 2023-01-30

**Authors:** Mahsa Mahjani, Sepehr Gohari, Hassan Ahangar

**Affiliations:** 1 Endocrine Research Center, School of Medicine, Shahid Beheshti University of Medical Sciences, Tehran, Iran; 2 Department of Family Medicine, Alborz University of Medical Science, Alborz, Iran; 3 Student Research Center, School of Medicine, Zanjan University of Medical Sciences, Zanjan, Iran; 4 Department of Cardiology, Mousavi Hospital, School of Medicine, Zanjan University of Medical Sciences, Zanjan, Iran

**Keywords:** Ultra-slow, Thrombolytic, Atrial Thrombus, COVID-19

## Abstract

The hyper-coagulopathy nature of COVID-19 is a prevalent consequence among patients. Free-floating right atrial thrombi are a relatively rare finding and the optimal therapy is a therapeutic dilemma.

We present a 37-year-old woman with acute dyspnea and fatigue. Several ground glass opacities were shown on computed tomography of chest that further proved to be associated with severe COVID-19 disease. A transthoracic echocardiography revealed a mobile right atrial mass with bilateral pulmonary embolism. She was considered high risk for surgical therapy by cardiovascular surgeons. She was then started on anticoagulation therapy for 5 days however the size regression of the thrombus remained unchanged. A regimen of low dose (24 mg) ultra-slow (24 h) intravenous infusion of alteplase, without bolus was initiated. Following the third day of thrombolytic therapy, the control echocardiography demonstrated complete resolution of the thrombus.

Prolonged infusion of low dose fibrinolytics can be an alternative treatment to surgery for right heart thrombi.

**Supplementary Information:**

The online version contains supplementary material available at 10.1186/s12959-023-00457-8.

## Introduction

Thromboembolic events are a major complication in COVID-19 patients. The widespread thrombosis events in COVID-19 patients despite prophylactic or fully therapeutic anticoagulation raise concerns regarding thrombotic adverse events specially in severely ill patients [1]. Detections of right heart thrombi (RHT) have increased with the extensive use of echocardiography [2]. Presence of free-floating intracardiac thrombus is an unusual finding that is associated with poor outcomes. Determining the most appropriate therapy for RHT with concurrent pulmonary embolism still remains a challenge [3]. Operative thrombectomy with exploration in right chambers is the conventional treatment, however surgery cannot be applicable to patients who are at high risk for bleedings, therefore novel systemic thrombolytic therapies are probable bailout approaches [4].

## Case presentation

**Fig. 1:**
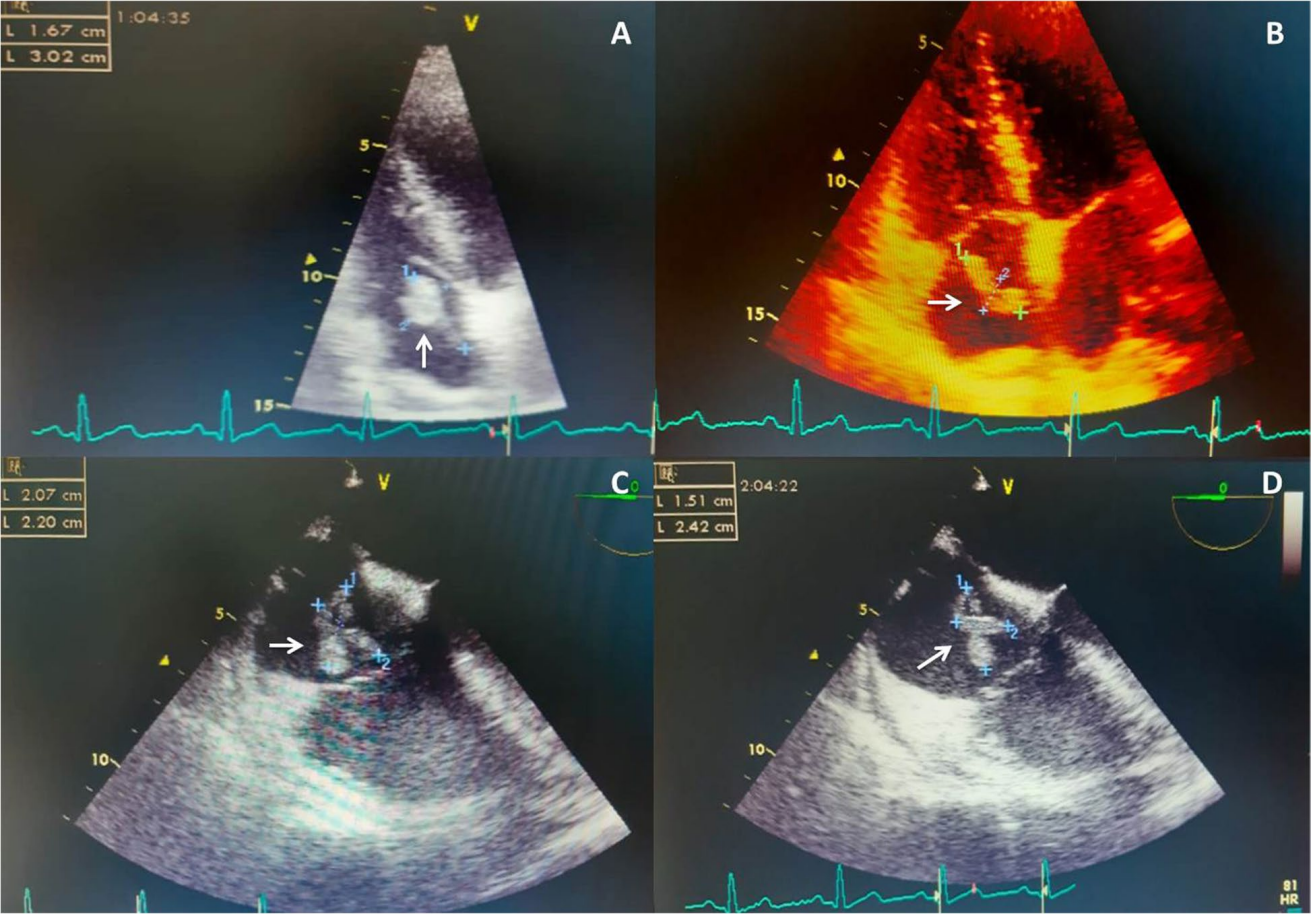
**A**, **B** Trans-thoracic echocardiography. **C**, **D** Trans-esophageal echocardiography. The imaging assessments showed a large lobulated mass (Clot) in right atrium attached to Chiari network (Arrow)

**Fig. 2:**
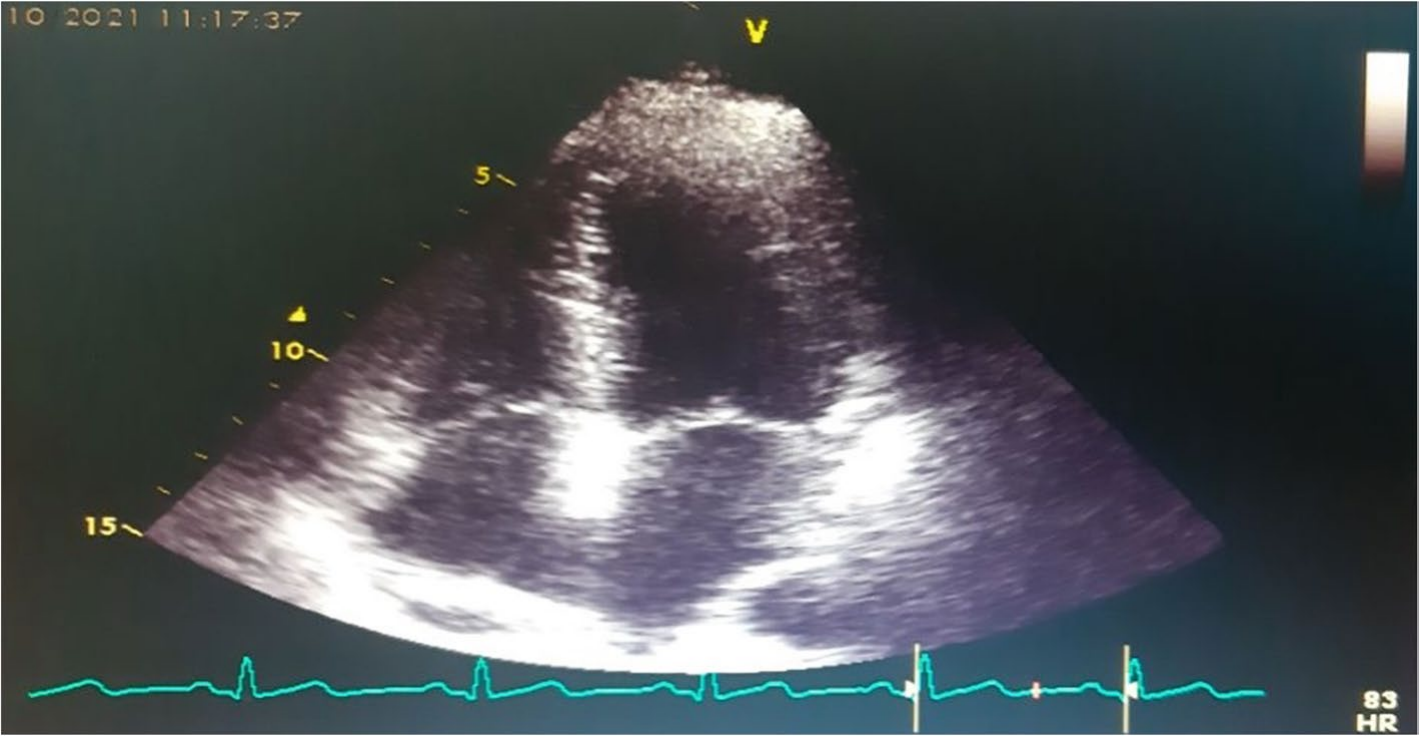
Epical view in transthoracic echocardiography after 72-h treatment showed complete resolution of thrombus

A 37-year-old female without known past medical history presented to emergency ward with a 5-day history of fatigue and dyspnea. She had tachypnea, respiratory rate of 31/min and oxygen saturation of 78%. A computed tomography (CT) of her chest entailed ground glass opacities. The lung CT scan manifestations were proved to be attributed to COVID-19 disease. On the 14^th^ day of admission, her respiratory failure persisted and she was admitted to intensive care unit (ICU) to receive non-invasive ventilation (NIV). Transthoracic echocardiography (TTE) had no abnormal findings on that date. On the 11^th^ day of ICU stay, a repeated bedside TTE revealed normal right and left ventricular function and elevated pulmonary pressure (PAP = 50 mmHg) and a large free-floating mass (2.6 × 1 cm) in right atrium. CT angiography on the same day demonstrated bilateral pulmonary embolism. No deep vein thromboses in pelvic or upper and lower extremities were detected on Doppler ultrasound. Cardiovascular surgeons considered her high risk for thrombectomy surgery. Despite anticoagulation therapy with heparin for 5 days, follow-up TTEs showed no significant resolution of thrombus. During this time, anticoagulants had to be held periodically in light of her gross hematuria episodes. She was then transferred to our hospital for further management. The TTE and transesophageal echocardiography (TEE) revealed a large free-floating mass (3.2 × 1.67 cm) in right atrium attached to prominent Chiari network and moving to the tricuspid valve with protrusions to right ventricle (Fig. 1, Additional file 1) and normal left/right ventricular function. Given her bleeding diathesis and clinical deterioration, rapid infusion of t-PA was not possible and thrombolytic therapy (TT) with ultra-slow intravenous infusion of low dose alteplase (1 mg/h) was planned. Following 24 h of TT, thrombus was partially regressed, however after 48 h of infusion, it had been significantly reduced in size, and therefore alteplase was continued. After 72 h of administrating thrombolytic, thrombus was completely dissolved (Fig. 2, Additional file 2). In the next 2 days, the patient was transferred to general ward and 7 days later was discharged from hospital with oral anticoagulants without any fibrinolytic related bleeding complications.

## Discussion

SARS-CoV-2 induces a prothrombotic state through various mechanisms [5]. Right ventricle dysfunction along with severe pulmonary artery hypertension contributes to the formation of right intracardiac thrombi. Adherent thrombi in right atrium usually encompasses benign prognosis; they are usually associated with implanted devices like prosthetic heart valves and develop in situ. The free-floating or so-called type “A” thrombi are worm-like shape and extremely mobile; they are the result of embolism in-transit that may cause cardiovascular collapse [6]. Such thrombi coexist almost always with acute pulmonary embolism (PE) [7]. Although RHT have occurred infrequently in COVID-19 patients, they are indicative of poor prognosis [8, 9]. Fluid analysis of confined COVID-19 pericardial involvement has exhibited elevated cytokine concentrations which are contributory factors to thrombosis [10]. TTE is the most used imaging modality for intracardiac masses, on account of its convenience and bedside availability. TEE is usually performed for additional direct visualization and distinguish thrombi from other right heart masses. Complicated massive PE with right atrial thrombi requires prompt emergency care because any delay to treatment can be lethal. Therapeutic options are comprised of urgent surgical embolectomy, anticoagulation and systemic or catheter-directed thrombolysis [11]. Surgery has its own sets of potential complications. Moreover, anticoagulation therapy alone is not recommended in very mobile and large thrombi since it may establish a systematic embolization state [12]. TT has been proposed as a non-invasive treatment and can be a suitable alternative therapy of choice because of its ability to accelerate clot lysis and pulmonary reperfusion, plus it renders simultaneous thrombolysis not only of cardiac but also of pulmonary arterial and femoral venous thrombi [13]. TT is indicted in massive PE. However, adverse effects of fibrinolysis including cerebral and non-cerebral hemorrhages are a matter of concern [14]. Macro- and micro-thrombosis in the course of COVID-19 disease can lead to detrimental neurological complications. Furthermore, there is an increased risk of eventual ischemic stroke subsequent to incomplete thrombolysis of cardiac thrombi [15].

Bolus and rapid doses of TT lead to accelerated destruction of thrombus which may be accompanied with higher rate of thromboembolic and bleeding events. The slow/ultra-slow protocols have been introduced in the last 2 decades as a novel strategy in the management of prosthetic mechanical valve thrombosis [16]. The rationale of further prolonging the infusion time is to reduce mortality and morbidity with preserved success rate. These current protocols are shown to have advantages over the classic TT [17].

The efficiency of low dose slow infusion in dissolving other thrombi not related to mechanical valves have been recently acknowledged in a few reports. A slow infusion (8 h) low dose (50 mg) t-PA regimen was successful in complete lysis of a mobile left ventricular thrombus within 24 h [18]. Administering of 25 mg rt-PA in 6 h was shown to be effective in lysis of thrombi in a patient with free-floating right atrium thrombus and in another patient with acute thrombotic left main coronary artery [19, 20]. Further, a study consists of 12 patients with thromboembolic events performed the slow infusion method of t-PA and revealed promising data regarding its safety and efficacy [21].

Therapeutic profile of low dose ultra-slow infusion thrombolytic therapy (LDUSITT) for thrombi without association to prosthetic valve has been barely discussed. There are only 3 case reports that had applied the LUDSITT protocol. One case had renal artery thromboembolism who was not eligible for percutaneous procedure and following the third dose of infusion, echocardiography showed no residue of the thrombus [22]. The other 2 cases were similar to our patient and presented with mobile thrombi in right atrium and received alteplase with a rate of 1 mg per hour, without bolus. Thrombi were thoroughly disappeared in both patients [23, 24] (Table 1).

**Table 1:** Case reports of right atrial thrombi treated with Low dose ultra-slow thrombolytic therapy

Study	age	gender	Comorbidities	Deep vein thrombosis	Pulmonary emboli	Characteristics of thrombi	Size of thrombi	Diagnostic imaging modality	Cause of switching to TT	Prior management of thrombi	Thrombolytic regimen	Duration of infusion	Complete resolution of thrombi	complications
Yilmaztepe et al	72	F	HTN	No	Yes	right atrial mobile mass moving with the tricuspid valve and dilated right ventricle	N/A	TTE	3th day post major surgery (high risk of bleeding)	None	IV 25 mg of rt-PA for 24 h, without bolus	18 h	Yes	Minor bleeding from surgery site
Alves Pinto et al	57	M	Mitral regurgitation, right ventricle dysfunction	Yes	Yes	two large mobile right atrial thrombi	2.4 × 1.5 cm and 3.6 × 3.7 cm	TTE	Refractory to anticoagulant therapy	Unfractionated heparin	IV 24 mg of alteplase for 24 h (1 mg per hour), without bolus	48 h	Yes	None
“This report”	37	F	COVID-19	No	Yes	Free-floating right atrial thrombi with protrusion from tricuspid valve to right ventricle	3.2 × 1.67 cm	TTE, TEE	Refractory to anticoagulant therapy, episodes of gross hematuria	Heparin	IV 24 mg of alteplase in 24 h (1 mg per hour), without bolus	72 h	Yes	None

*HTN* Hypertension, *TTE* Trans-thoracic echocardiography, *IV* Intravenous, *TEE* Trans-esophageal echocardiography, *F* Female, *M* Male

In our case, the free-floating thrombus was regarded as type A thrombi via imaging features and remained refractory despite anticoagulation therapy. The patient was a high-risk candidate for surgery. Her hyper-coagulopathy state secondary to COVID-19 was probably responsible in the development of such large thrombus. Due to imminent circulatory collapse a regimen of low dose ultra-slow alteplase 24 mg in 24 h without bolus was initiated. After 72 h (totally 72 mg) of infusion, complete resolution of thrombus was achieved with no hemorrhagic complications. Although fibrinolytics are contraindicated in active bleedings, the use of low dose ultra-slow alteplase in such fatal condition was an opportune and life-saving choice of treatment.

## Conclusion

Altogether the available scarce evidence advocates the implication of LDUSITT in RHT in patients with refractory response to classical treatment and higher bleeding risk. Additional studies are warranted to confirm the protocol as an alternative therapeutic approach when surgery is not feasible. Moreover, these findings highlight the use of bedside transthoracic echocardiography as a valuable assessment and screening tool in patients with severe COVID-19.

## Supplementary Information

**Additional file 1.**

**Additional file 2.**

## Data Availability

Not applicable.

## Abbreviations

LDUSITT: Low dose ultra-slow infusion thrombolytic therapy
RHT: Right heart thrombi
CT: Computed Tomography
ICU: Intensive care unit
NIV: Non-invasive ventilation
TTE: Transthoracic echocardiography
TEE: Transesophageal echocardiography
TT: Thrombolytic therapy
PE: Pulmonary embolism
